# Association of body mass index with knee cartilage damage in an asymptomatic population-based study

**DOI:** 10.1186/s12891-017-1884-7

**Published:** 2017-12-08

**Authors:** Alvin Keng, Eric C. Sayre, Ali Guermazi, Savvakis Nicolaou, John M. Esdaile, Anona Thorne, Joel Singer, Jacek A. Kopec, Jolanda Cibere

**Affiliations:** 1 University of Toronto, Toronto, ON Canada; 2 Arthritis Research Centre of Canada, Richmond, BC Canada; 3 Section of Musculoskeletal Imaging, Boston University Medical Center, Boston, MA USA; 4 Boston University School of Medicine, Boston, MA USA; 5 Vancouver General Hospital, Vancouver, BC Canada; 6 Department of Medicine, University of British Columbia, Vancouver, BC Canada; 7 Canadian HIV Trials Network, Vancouver, BC Canada; 8 School of Population and Public Health, University of British Columbia, Vancouver, BC Canada; 9 Arthritis Research Canada Milan Ilich Arthritis Research Centre, 5591 No. 3 Road, Richmond, BC V6X2C7 Canada

**Keywords:** Obesity, Cartilage, Knee, Magnetic resonance imaging, Population-based, Asymptomatic

## Abstract

**Background:**

Cartilage changes are an important early finding of osteoarthritis (OA), which can exist even before symptoms. Our objective was to determine the prevalence of knee cartilage damage on magnetic resonance imaging (MRI) in an asymptomatic population-based cross-sectional study and to evaluate the association of body mass index (BMI) with cartilage damage.

**Methods:**

Subjects, aged 40-79 years, without knee pain (*n* = 73) were recruited as a random population sample and assessed for BMI (kg/m^2^), including current BMI (measured), past BMI at age 25 (self-reported) and change in BMI. Knee cartilage was scored semi-quantitatively (grades 0-4) on MRI. In primary analysis, cartilage damage was defined as ≥2 (at least moderate) and in a secondary analysis as ≥3 (severe). We also conducted a sensitivity analysis by dichotomizing current BMI as <25 vs. ≥25. Logistic regression was used to evaluate the association of each BMI variable with prevalent MRI-detected cartilage damage, adjusted for age and sex.

**Results:**

Of 73 subjects, knee cartilage damage ≥2 and ≥3 was present in 65.4% and 28.7%, respectively. The median current BMI was 26.1, median past BMI 21.6, and median change in BMI was a gain of 2.8. For cartilage damage ≥2, current BMI had a non-statistically significant OR of 1.65 per 5 units (95% CI 0.93-2.92). For cartilage damage ≥3, current BMI showed a trend towards statistical significance with an OR of 1.70 per 5 units (95% CI 0.99-2.92). Past BMI and change in BMI were not significantly associated with cartilage damage. Current BMI ≥ 25 was statistically significantly associated with cartilage damage ≥2 (OR 3.04 (95% CI 1.10-8.42)), but not for ≥3 (OR 2.63 (95% CI 0.86-8.03)).

**Conclusions:**

MRI-detected knee cartilage damage was highly prevalent in this asymptomatic population-based cohort. We report a trend towards significance of BMI with cartilage damage severity. Subjects with abnormal current BMI (≥25) had a 3-fold increased odds of cartilage damage ≥2, compared to those with normal BMI. This study lends support towards the role of obesity in the pathogenesis of knee cartilage damage at an asymptomatic stage of disease.

## Background

Diagnosing knee osteoarthritis (OA) has traditionally relied on a combination of clinical symptoms (knee pain, crepitus, stiffness) and radiograph findings (osteophytosis and joint space narrowing). Recently, MRI has been used extensively in the evaluation of OA. We previously reported that MRI-based knee cartilage damage was highly prevalent in symptomatic subjects despite absent radiographic changes [1]. Similar studies for asymptomatic cohorts have not provided consistent prevalence rates [2–8].

MRI-related research to date has focused primarily on symptomatic individuals [9]. However, asymptomatic individuals may have undetected cartilage damage that places them at risk for developing symptomatic OA. For prevention of knee OA to be successful, early recognition and diagnosis of these individuals is critical and a better understanding of risk factors for cartilage damage in asymptomatic individuals is important. Body mass index (BMI) is well-established as a risk factor for the development of symptomatic cartilage damage and OA [9]; however, fewer studies are available on its relationship to asymptomatic cartilage damage.

In our population-based cross-sectional study, our objective was to determine the prevalence of MRI-detected cartilage damage in subjects without knee pain, and to evaluate the association of current BMI, past BMI and change in BMI with asymptomatic cartilage damage. In doing so, we hope to further our understanding of factors leading to cartilage damage before the onset of OA.

## Methods

### Participants

This is a cross-sectional study of a population-based sample of people without knee pain. Recruitment has been described previously in detail [10] and, with the exception of knee pain, was identical to that of our symptomatic knee cohort [1]. This asymptomatic cohort was recruited from the same population as the symptomatic cohort in the Greater Vancouver area, using an identical multi-stage protocol. Briefly, invitation letters were mailed to a randomly generated list of households (*n* = 4300), followed by a standardized telephone screening protocol. Of the 2355 English-speaking people who were reached by telephone, 1091 (46.3%) agreed to participate in the screening survey. Of these, 104 (9.5%) met the eligibility criteria and were invited to attend the study center, at which time a further 31 individuals were excluded, resulting in a cohort of 73 subjects with complete assessments. Recruitment was conducted using stratified sampling to achieve equal representation within age decades and sex. One knee was used per participant and selected at random. Inclusion criteria were: between 40 and 79 years old and a ‘no’ response to the following two pain questions: 1) have you had pain, aching or discomfort in or around either knee on most days of the month at any time in the past, 2) have you had any pain, aching or discomfort in or around either knee during the last year. Exclusion criteria were: 1) inability to undergo radiography or MRI, 2) history of prior knee arthroplasty, 3) diagnosis of fibromyalgia or inflammatory arthritis, 4) knee injury or surgery within the last 6 months. Our study was conducted in accordance with the Declaration of Helsinki and was approved by the Clinical Research Ethics Board, University of British Columbia (ACE-KOA: H07-00793). All subjects gave their written informed consent.

### MR image acquisition

MRI was specified to be performed within a month of the clinic visit, using the same magnet and identical imaging protocol as for the symptomatic knee cohort study. We have previously described the protocol in detail [1, 10, 11]. Briefly, MRI was performed on a General Electric 1.5 T magnet (General Electric Medical Systems, Milwaukee, WI). Four MRI sequences were obtained, including a fat-suppressed T1-weighted 3-dimensional spoiled gradient-recalled acquisition in the steady state sequence with images obtained in the sagittal plane and reformat images in the axial and coronal planes, a fat-suppressed T2-weighted fast spin-echo (FSE) sequence with images obtained in the coronal plane, a T1-weighted FSE sequence with images obtained in the oblique sagittal plane and a T2-weighted FSE sequence with images obtained in the oblique sagittal plane.

### MRI semi-quantitative scoring

Six knee joint surfaces were assessed, including the medial tibia, lateral tibia, medial femur, lateral femur, patella, and trochlear groove [1, 11]. MR cartilage (MRC) was graded on a semi-quantitative scale of 0–4 based on the following definitions, as previously described by Disler et al. [12]: 0 = normal, 1 = abnormal signal without a cartilage contour defect, 2 = contour defect of <50% cartilage thickness, 3 = contour defect of 50–99% cartilage thickness, and 4 = 100% cartilage contour defect with subjacent bone signal abnormality. The MRC score for each subject was determined using the worst cartilage lesion of any of the 6 regions. The MR images were read by a single experienced musculoskeletal radiologist (AG), who was blinded to the radiographic and clinical information and with excellent intrarater reliability of cartilage readings, ranging from 0.84 to 1.00, as previously described [1, 11].

### Radiographic assessment

Knee radiography was obtained identical to the symptomatic knee cohort study and has been described in detail previously [1, 11]. Briefly, fixed-flexion knee radiographs were obtained with the SynaFlexer™ positioning frame [13] and a skyline view in the supine position. Radiographs were scored independently by 2 readers (JC, SN), blinded to clinical and MRI information, using Kellgren Lawrence (K/L) grading (scale 0–4), with adjudication of differences by consensus. The interrater reliability has previously been reported to be good, with an intraclass correlation coefficient of 0.79 [1, 11].

### Clinical evaluation

Subjects completed a comprehensive questionnaire to assess demographics and OA risk factors. Self-reported past weight and height at age 25 was ascertained. Current weight and height were measured by a single examiner. Weight was measured on a balance beam scale to the nearest pound. Height was measured to the nearest eighth of an inch. BMI was calculated as weight [kg]/height squared [m^2^]. Change in BMI was calculated as the difference between current BMI and past BMI at age 25.

### Statistical analysis

Data was summarized using frequencies, means (with SDs), or medians (range), as appropriate. Logistic regression analysis was performed, adjusted for current age and sex, to evaluate the association of each of the predictor variables (current BMI, past BMI, and change in BMI) with prevalent MRI-detected cartilage damage. BMI was treated as a continuous variable and its effect was reported as a 5-unit change. We used 5-unit increments because such an increase generally represents a change in BMI category and has clinical utility. We conducted an additional sensitivity analysis by dichotomizing current BMI into <25 versus ≥25. In the primary analysis, cartilage damage was defined as an MRC score of ≥2 at any joint site (at least moderate cartilage damage). A secondary analysis was performed defining cartilage damage as an MRC score of ≥3 at any joint site (severe cartilage damage).

To obtain population-based estimates, all analyses were performed using age decade-sex stratum sampling weights and were performed using SAS version 9.3.

## Results

Seventy-three asymptomatic subjects (one knee per subject) were included in our study. Demographic and clinical variables are shown in Table 1. Median age was 52.0 years and 56.5% were women. The distribution of cartilage damage from grades 0 to 4 were 34.0%, 0.6%, 36.7%, 21.5%, and 7.2%, respectively. As such, the majority of subjects in our asymptomatic cohort, 65.4%, had MRI-detected at least moderate cartilage damage (≥2). Severe cartilage damage (≥3) was seen in 28.7%. KL grade 0 was present in 52.7%, KL grade 1 in 39.8%, while 7.5% had radiographic OA (ROA) (5% KL grade 2 and 2.5% KL grade 3). The distribution of MRI scores by KL grade is shown in Table 2. Median current BMI was 26.1 kg/m^2^ [interquartile range = 22.8-29.2]. 43.4% of individuals had a current BMI < 25, and 56.6% had a BMI ≥ 25. Median past BMI at age 25 was 21.6 kg/m^2^ [interquartile range = 20.8-23.9] and median change in BMI was 2.8 kg/m^2^ [interquartile range = 1.3-6.5]. The distribution of cartilage damage by joint site was as follows: medial femoral condyle 32.6%, medial tibial plateau 8.1%, lateral femoral condyle 20.8%, lateral tibial plateau 10.1%, patella 36.9%, trochlear groove 49.4%.

**Table 1:** Demographic and clinical characteristics of study population

	*n* = 73
Female, n (%)	41.3 (56.5)
Age, years	52.0 (47.0-60.0)
KL grade 0, n (%)	38.5 (52.7)
KL grade 1, n (%)	29.0 (39.8)
KL grade 2, n (%)	3.7 (5.0)
KL grade 3, n (%)	1.8 (2.5)
KL grade 4, n (%)	0 (0)
Current BMI	26.1 (22.8-29.2)
Current BMI <25, n (%)	31.7 (43.4)
Current BMI ≥25, n (%)	41.3 (56.6)
Past BMI at age 25	21.6 (20.8-23.9)
Change in BMI	2.8 (1.3-6.5)
MRC score 0, n (%)	24.8 (34.0)
MRC score 1, n (%)	0.5 (0.6)
MRC score 2, n (%)	26.8 (36.7)
MRC score 3, n (%)	15.7 (21.5)
MRC score 4, n (%)	5.2 (7.2)

All numbers are medians (interquartile range) unless otherwise indicated

Stratum-sampling weights were used, hence n = weighted counts which are non-integer (see Methods)

*MRC* magnetic resonance cartilage, *KL* Kellgren Lawrence, *BMI* body mass index

**Table 2:** Distribution of MRC Scores within KL Grade, n (%)

	MRC 0	MRC 1	MRC 2	MRC 3	MRC 4	TOTAL *n* = 73
KL 0	12.5 (17.1)	0 (0)	17.1 (23.4)	4.8 (6.6)	4.1 (5.6)	38.5 (52.7)
KL 1	9.8 (13.4)	0.5 (0.6)	8.5 (11.7)	9.1 (12.5)	1.2 (1.6)	29.0 (39.8)
KL 2	1.3 (1.8)	0 (0)	1.2 (1.7)	1.2 (1.6)	0 (0)	3.7 (5.0)
KL 3	1.2 (1.7)	0 (0)	0 (0)	0.6 (0.8)	0 (0)	1.8 (2.5)
KL 4	0 (0)	0 (0)	0 (0)	0 (0)	0 (0)	0 (0)

We used stratum-sampling weights based on recruitment numbers, hence (weighted) counts are non-integer

*MRC* magnetic resonance cartilage, *KL* Kellgren Lawrence

In primary analyses, current BMI showed a non-statistically significant OR of 1.65 (95% CI 0.93-2.92, per 5 units) with at least moderate cartilage damage (≥2), adjusted for age and sex. (Table 3). We also did not observe a statistically significant association of past BMI (OR 1.70, 95% CI 0.69-4.21, per 5 units) and change in BMI (OR 1.53, 95% CI 0.81-2.88, per 5 units) with cartilage damage ≥2.

**Table 3:** Association of BMI with at least moderate MRI-detected cartilage damage (MRC ≥ 2) using logistic regression analysis

Clinical variables	Crude OR (95% CI)	Adjusted OR^a^ (95% CI)
Current BMI (per 5 units)	1.75 (0.98-3.12)	1.65 (0.93-2.92)
Current BMI ≥ 25 vs. <25	3.14 (1.16-8.53)	3.04 (1.10-8.42)
Past BMI (per 5 units)	1.59 (0.71-3.54)	1.70 (0.69-4.21)
Change in BMI (per 5 units)	1.48 (0.80-2.72)	1.53 (0.81-2.88)

*BMI* body mass index

^a^adjusted for age and sex

In secondary analyses, evaluating the association of our BMI variables with severe cartilage damage (≥3), we found similar results (Table 4). In unadjusted analysis, current BMI was significantly associated with severe cartilage damage ≥3 (OR 1.74, 95% CI 1.01-3.00, per 5 units) although, after adjustment for age and sex, this was only of borderline statistical significance (OR 1.70, 95% CI 0.99-2.92, per 5 units). We did not observe a statistically significant association of past BMI (OR 2.24, 95% CI 0.90-5.56, per 5 units) or change in BMI (OR 1.41, 95% CI 0.75-2.63, per 5 units) with cartilage damage ≥3.

**Table 4:** Association of BMI with severe MRI-detected cartilage damage (MRC ≥ 3) using logistic regression analysis

Clinical variables	Crude OR (95% CI)	Adjusted OR^a^ (95% CI)
Current BMI (per 5 units)	1.74 (1.01-3.00)	1.70 (0.99-2.92)
Current BMI ≥ 25 vs. <25	2.74 (0.91-8.24)	2.63 (0.86-8.03)
Past BMI (per 5 units)	1.79 (0.81-3.94)	2.24 (0.90-5.56)
Change in BMI (per 5 units)	1.44 (0.82-2.54)	1.41 (0.75-2.63)

*BMI* body mass index

^a^adjusted for age and sex

In additional sensitivity analyses, subjects with a current BMI ≥25, compared to those with BMI <25, had a statistically significantly increased odds of cartilage damage ≥2 with OR 3.04 (95% CI 1.10-8.42), adjusted for age and sex (Table 3). For cartilage damage ≥3, the result was similar in magnitude although not statistically significant (OR 2.63 (95% CI 0.86-8.03)) (Table 4).

## Discussion

In this population-based study of asymptomatic middle-aged and elderly people, we report four key findings on MRI-based cartilage damage: prevalence, a statistically non-significant trend towards an association with current BMI, and no association with past BMI and change in BMI.

### Prevalence

Firstly, MRI-detected cartilage damage was highly prevalent, with 65.4% having at least moderate cartilage damage (≥2) and 28.7% having severe cartilage damage (≥3). Past literature on asymptomatic individuals has reported a prevalence ranging from 11.4% to 79% [2–8]. Our study agrees with most studies that cartilage damage is highly prevalent among middle-aged and older adults without knee pain [2–7]. Of note, the one study that reported a low prevalence of 11.4% included much younger participants (as low as 20 years of age) and used a 1.0 T MRI, which may have resulted in a lower prevalence [8]. Thus, we can extrapolate an estimated prevalence between 53.5-79% from the remaining studies, which include populations from the Melbourne Collaborative Cohort Study and the Osteoarthritis Initiative (OAI), which used a 3 T MRI. We add to the current literature by including one of the widest age range of participants (40-79 years old). In addition, we have used a more rigorous definition for cartilage damage: as strictly a contour defect, in contrast to some prior studies which included early signal irregularities. We also present data on the prevalence of severe cartilage damage, reported at 28.7% and information comparing MRI-based damage with radiographic findings. In contrast to our MRI data, we reported ROA (KL grade ≥ 2) in only 7.5% of our cohort. Furthermore, when we look at the distribution of MRC scores within KL grade, while 52.7% of subjects have a KL score of 0, only 17.1% of individuals had both a KL grade of 0 and MRC score of 0, indicating that most individuals had some degree of cartilage damage, despite showing no signs on radiograph. These discrepancies highlight the principle that MRI detects joint damage well before the development of radiographic changes or symptoms, in keeping with what we established in our symptomatic population [1].

### Current BMI

We report that a 5-unit increase in current BMI had a non-statistically significant 1.7-fold increased odds of MRI-based moderate and severe cartilage damage, although there was a trend towards statistical significance. A 5-unit increase in BMI is a clinically meaningful measure, since it means that a person would have changed into a higher BMI category, (e.g. going from normal to overweight or from overweight to obese). We also found a statistically significant 3-fold increased odds of cartilage damage in people with abnormal current BMI (BMI ≥25) compared to those with normal BMI, adding strength to the idea that increased weight is linked to cartilage damage, even in asymptomatic people. In symptomatic populations, the relationship between BMI and cartilage damage or OA has been elucidated to a much greater extent. Mezhov et al. (2014) [9] reviewed 22 studies that examined the relationship between obesity and knee cartilage in patients with knee OA, concluding that there was a detrimental effect of BMI and fat mass on cartilage, though cohort studies were relatively lacking. In comparison, this is less clear in asymptomatic studies. Our finding of an increased odds of cartilage damage with BMI, although short of statistical significance, is in keeping with most [3, 4, 7, 14–16], but not all studies [2, 17]. Among the six studies reporting an association, only one included both tibiofemoral and patellar cartilage defects [7], similar to ours. They studied 137 volunteers from the OAI without radiographic OA and reported cross-sectionally that cartilage damage was significantly more common in overweight and obese subjects compared to normal BMI subjects, and longitudinally, new or worsening cartilage lesions were significantly higher in obese subjects after 36 months [7]. Their findings agree with our finding of an increased odds of threefold for patients with a BMI ≥ 25. However, in contrast to our study, they included a highly selected cohort of subjects at risk of OA. In addition, their definition of no knee pain included individuals with some knee symptoms who would have been excluded in our study. Other positive studies either examined only tibiofemoral cartilage [3, 4] or only patellar cartilage [14–16]. Brennan et al. (2010) [3] reported an association between current BMI and prevalent tibiofemoral cartilage defects with an adjusted OR of 1.07 per one unit of BMI increase, which translates to an OR of 1.4 per 5 units of BMI increase. This finding is comparable to our study’s result of OR 1.65 per 5 units. Our study contributes to the literature in two ways. Firstly, our population-based study allows for more generalizability. Among these six positive studies, only two were population-based and they studied a younger (age 30-49) and only female population [3, 14], whereas we included a wide age range (40-79 years) and both males and females. Secondly, our study is the first to examine and report on a trend to significance with a more severe grading of cartilage damage. In contrast, two studies did not find an association between BMI and prevalent cartilage damage [2, 17]. Guermazi et al. (2012) [2] stratified 710 participants into normal, overweight, and obese and found no differences in prevalence of tibiofemoral cartilage defects. In contrast to our study, they used a mixed population aged >50 years old with no radiographic evidence of knee OA with 29% experiencing some knee pain in the last month. While the other study by Berry et al. (2010) [17] did not find an association between tibiofemoral cartilage defects and BMI, they did, however, establish an association with fat mass measured by dual X-ray absorptiometry. BMI is a crude, but clinically useful, measure of obesity. Although fat mass is a different marker of obesity, their finding parallels our study by suggesting an impact of obesity or adiposity on cartilage health in asymptomatic individuals.

### Past BMI

We did not find a statistically significant association of past BMI with cartilage damage. Four previous studies have established a relationship between past BMI and cartilage damage [4, 7, 14, 15]. In contrast to our study, which relied on self-reported data, these studies used measured data. Furthermore, most studies used an interval of 10 years for past BMI with the exception of one [7] which used BMI 36 months prior, whereas the time interval for our subjects was greater (about 20-30 years more). One other population-based study by Brennan et al. (2010) [3] did not find such an association with past BMI measured 10 years prior and prevalent tibiofemoral cartilage defects. Of note, no studies have looked at a past BMI greater than 10 years prior, likely given that a longer time frame is challenging to study. Understanding the impact of BMI in early adulthood, as we had done, would be clinically useful. However, a measured variable would have been ideal as self-reported data increases variability.

### Change in BMI

No studies have found an association of change in BMI with cartilage damage, similar to our findings. The median increase in BMI of 2.8 kg/m^2^ in our cohort was rather modest and may not have been sufficient to detect an effect. Furthermore, as BMI at age 25 was self-reported, it may be inaccurate in relation to the measured current BMI. Nevertheless, our finding is in keeping with the literature. Even studies that reported an association between prevalent cartilage damage and current BMI did not find an association with change in BMI [3, 4, 14]. Change in BMI was also very modest in these studies with the mean change ranging from 0.7-2.3 kg/m^2^, and studied over a limited time frame of 10 years. In fact, our study had the largest mean change and the longest time span. Greater changes in BMI may have an impact on cartilage damage but requires further study.

Our study is limited by its small sample size, which may have affected our power and reduced our ability to detect weaker associations. Nevertheless, despite our small sample size, we found an increased odds ratio of current BMI with cartilage damage with borderline statistical significance for severe cartilage damage and a statistically significant association of BMI ≥25 with moderate cartilage damage. Our study is cross-sectional. This limits our ability to establish causality. Longitudinal studies will be required to evaluate the causal link between BMI and cartilage damage in asymptomatic cohorts. Furthermore, since asymptomatic cartilage damage is pre-clinical, longitudinal studies will be required to establish whether this stage of disease progresses to future symptomatic or radiographic disease.

## Conclusion

In conclusion, in our population-based study of people without knee pain, MRI-detected cartilage damage was highly prevalent. We found a trend towards statistical significance for an association between 5-unit increase in current BMI and cartilage damage with OR of 1.65 and 1.70 for cartilage damage ≥2 and ≥3, respectively. Furthermore, subjects with an abnormal current BMI (≥25) had a statistically significantly 3-fold increased odds of cartilage damage ≥2, compared to those with normal BMI. This study lends support towards the role of obesity in the pathogenesis of cartilage damage at an asymptomatic stage of disease and increases our understanding of OA. Further studies are needed to establish whether a threshold relationship exists between BMI and cartilage damage, and whether weight maintenance or weight loss reduces the risk of future cartilage damage or symptomatic OA.

## Abbreviations

BMI: Body mass index
KL: Kellgren Lawrence
MRC: MR Cartilage
MRI: Magnetic resonance imaging
OA: Osteoarthritis
OAI: Osteoarthritis Initiative
ROA: Radiographic Osteoarthritis
